# Elucidating three-way interactions between soil, pasture and animals that regulate nitrous oxide emissions from temperate grazing systems

**DOI:** 10.1016/j.agee.2020.106978

**Published:** 2020-09-15

**Authors:** G.A. McAuliffe, M. López-Aizpún, M.S.A. Blackwell, A. Castellano-Hinojosa, T. Darch, J. Evans, C. Horrocks, K. Le Cocq, T. Takahashi, P. Harris, M.R.F Lee, L. Cardenas

**Affiliations:** a Rothamsted Research, North Wyke, Okehampton, Devon, EX20 2SB, UK; b University of Florida, IFAS Southwest Florida Research and Education Center, Immokalee, FL, 34142, USA; c University of Bristol, Bristol Veterinary School, Langford, Somerset, BS40 5DU, UK

**Keywords:** Nitrous oxide, Beef cattle, Urine, Dung, Climate change, Denitrification, Nitrification, Soil microbial communities

## Abstract

Pasture-based livestock farming contributes considerably to global emissions of nitrous oxide (N_2_O), a powerful greenhouse gas approximately 265 times more potent than carbon dioxide. Traditionally, the estimation of N_2_O emissions from grasslands is carried out by means of plot-scale experiments, where externally sourced animal excreta are applied to soils to simulate grazing conditions. This approach, however, fails to account for the impact of different sward types on the composition of excreta and thus the functionality of soil microbiomes, creating unrealistic situations that are seldom observed under commercial agriculture. Using three farming systems under contrasting pasture management strategies at the North Wyke Farm Platform, an instrumented ruminant grazing trial in Devon, UK, this study measured N_2_O emissions from soils treated with cattle urine and dung collected within each system as well as standard synthetic urine shared across all systems, and compared these values against those from two forms of controls with and without inorganic nitrogen fertiliser applications. Soil microbial activity was regularly monitored through gene abundance to evaluate interactions between sward types, soil amendments, soil microbiomes and, ultimately, N_2_O production. Across all systems, N_2_O emissions attributable to cattle urine and standard synthetic urine were found to be inconsistent with one another due to discrepancy in nitrogen content. Despite previous findings that grasses with elevated levels of water-soluble carbohydrates tend to generate lower levels of N_2_O, the soil under high sugar grass monoculture in this study recorded higher emissions when receiving excreta from cattle fed the same grass. Combined together, our results demonstrate the importance of evaluating environmental impacts of agriculture at a system scale, so that the feedback mechanisms linking soil, pasture, animals and microbiomes are appropriately considered.

## Highlights

• Urinary nitrogen concentrations were lowest on animals consuming high sugar grasses.
• However, soil under high sugar grasses recorded the highest N_2_O emissions.
• Synthetic urine generated N_2_O emissions inconsistent with locally collected urine.
• Differences in emissions amongst systems were explained by gene abundance ratios.
• Results indicate the importance of soil-pasture-animal-microbiome interactions.

## 1 Introduction

Agriculture is one of the greatest contributors to emissions of nitrous oxide (N_2_O) (Galloway et al., 2004; Reay et al., 2012), a key greenhouse gas (GHG) approximately 265 times more potent than carbon dioxide (IPCC, 2013) with an atmospheric residence time of around 116 years (Prather et al., 2015). Globally, the majority of N_2_O emissions arising from agricultural production occur due to livestock manure left on pasture (36 %) and the application of synthetic fertilisers (28 %) (FAO, 2016a). As farmlands occupy 37 % of the Earth’s land surface and grasslands account for 67 % of that area (FAO, 2016b), there is a clear and urgent need to reduce N_2_O emissions that originate from grazing livestock production systems (McAuliffe et al., 2018a). This case is particularly strong as current efforts to mitigate climate impacts of agriculture do not match up against that of many other industries. In the UK, for example, the agricultural sector reduced N_2_O emissions by ∼16 % between 1990 and 2013; during the same period, the industrial processing sector achieved a ∼50 % reduction (DECC, 2015).

In grazing livestock systems, N_2_O is primarily produced from soil transformation processes such as nitrification and denitrification of nitrogen (N) from inorganic (i.e. manufactured) fertiliser and organic sources such as excreta deposited on pastures during grazing or applied as manure. Livestock excreta is high in N content as typically only a small fraction (5–30 %) of protein and non-protein N in animal feed is retained in milk, meat and eggs, with the remaining component largely lost via urine and dung (Oenema et al., 2005). It can therefore be hypothesised that the more N retained in the animal, the less N deposited onto the soil, ultimately resulting in lower N_2_O emissions. This concept has led to an emerging framework to associate N_2_O emissions with nitrogen use efficiency (NUE) of agricultural systems (EU Nitrogen Expert Panel, 2015; Cardenas et al., 2019), whereby changes in feeding regimes, e.g. utilisation of legumes (Marley et al., 2007) and grasses with a high level of available water soluble carbohydrates (WSC) (Lee et al., 2002; Edwards et al., 2007), are highlighted as potential mitigation strategies.

The estimation of N_2_O emission factors (EFs), however, is seldom carried out at a system scale due, amongst other reasons, to practical constraints surrounding field experiments. Despite strong evidence supporting causal dietary effects on N partitioning and excretion (Parsons et al., 2011; Cheng et al., 2017), urine and dung for plot treatments are frequently collected from a herd external to the pasture on which emissions are actually measured. Such “outsourcing” has the scientific merit of standardising, and therefore theoretically eliminating, the fixed effect attributable to individual animals, especially when trials are carried out across multiple sites (van der Weerden et al., 2011). Nonetheless, EFs derived in this manner are likely to be biased, as neither animal, soil nor sward effects are completely accounted for (Simon et al., 2019).

Furthermore, environmental burdens assessed through plot experiments tend to overlook farming practices that are commonplace in commercial agriculture (Takahashi et al., 2018). Arguably the most archetypical example in the present context is the application of synthetic N fertilisers to non-leguminous swards, a routine task performed across a large area of temperate grasslands (Buckingham et al., 2014). Nevertheless, a recent review of 81 studies encompassing 254 unique urine treatments from around the world (López-Aizpún et al., 2020) was unable to identify any single case where the dosage of synthetic fertiliser was determined to mirror local farmers’ practices (e.g. incorporating split applications) or, even more crucially, adjusted according to the presence or absence of N-fixing legumes in the sward (Hoogendoorn et al., 2016). Such discrepancies between science and practice are paradoxical and undesirable because, by definition, EFs are designed to facilitate policy debates for climate change mitigation (Saggar et al., 2015; Chadwick et al., 2018). For this reason, IPCC guidelines explicitly acknowledge the need to make EFs as site-specific and system-specific as practically possible (IPCC, 2019).

System-scale EF measurements also have the potential to enhance current understanding of biochemical mechanisms regulating the N-cycle in grassland soils through the abundance of functional marker genes that represent particular enzymes or functional communities. Soil microbial communities play a crucial role in nutrient cycling and nutrient availability of grassland systems: nitrification, the aerobic oxidation of ammonium (NH_4_^+^) to nitrate (NO_3_^−^), is commonly represented by the enzymes ammonia monooxygenase encoded by the *amoA* gene of ammonia-oxidising archaea (*amoA* AOA) and bacteria (*amoA* AOB) (Rotthauwe et al., 1997). Denitrification, the sequential reduction of NO_3_- to gaseous nitrogen (N_2_) under oxygen-limiting conditions, is commonly represented by NO_3_^−^ and N_2_O reductase enzymes encoded by the *nirK*/*nirS*, and *nosZ*I*/nosZ*II genes, respectively. As these soil microbial communities are likely to be affected by a change in aboveground plant communities both directly through soil water/nutrient availability and indirectly through the chemical composition of excreta, examining their abundances alongside soil composition, pasture quality and GHG fluxes could improve identification of “hotspots” within the N-cycle associated with on-farm interventions. This question is especially pertinent to current climate policy debates surrounding GHG emissions from soils, where knowledge on the structure and composition of prokaryotic and eukaryotic communities in grassland soils is limited, as is information on factors affecting the abundance of N-cycling genes (Kaiser et al., 2016).

The overarching aim of the present study, therefore, was to compare direct N_2_O emissions arising from three different pasture types commonly used in temperate grasslands under an experimental setting that is as similar to commercial agriculture as possible, so that biochemical interactions within each closed system are appropriately and realistically incorporated. To achieve this goal, a field trial was designed to evaluate: (1) the impact of different sward management strategies on faeces and urine composition; (2) the impact of urine/dung compositions on N losses from the soil; (3) the abundance of N-cycle regulating functional communities under different sward management strategies and different urine/dung compositions; and (4) “all-inclusive” EFs more representative of the farming systems studied. The trial was carried out under the strict principle that the closed nutrient cycle that represents each farming system would be maintained, with all excreta collected from animals returned to the same pasture they were grazing.

## 2 Materials and methods

### 2.1 Study site

The North Wyke Farm Platform (NWFP; 50°45′N, 3°50′W), a UK National Capability managed by Rothamsted Research, comprises three hydrologically isolated small-scale grazing livestock farms known as “farmlets”, each of which is divided into 7 fields. Each farmlet maintains 30 weaned beef cattle as well as 75 ewes and their lambs (Orr et al., 2016, 2019) and occupies approximately 21 ha under the following pasture management strategies:

1 Permanent pasture (PP), predominately composed of perennial ryegrass (*Lolium perenne*) with some unsown grass, legume and forb species. It receives N fertiliser at a standard rate in the region (Section 2.2). None of the seven fields has been ploughed for at least 20 years.
2 White clover (*Trifolium repens*)/perennial ryegrass mix (WC), aiming to maintain 30 % ground cover by the former. No N fertiliser is used due to clover’s atmospheric N fixation. The perennial ryegrass variety is identical to that sown on monoculture fields (below; HS).
3 Perennial ryegrass monoculture (HS), utilising an innovative high sugar variety (*Lolium perenne* cv. AberMagic). Similar to PP, this system receives N fertiliser at a standard rate.

All cattle are born and reared at a cow-calf enterprise adjacent to the study site until weaning, and then randomly assigned to the three systems. This allocation is carried out through a covariate-based constrained randomisation process, which is designed to achieve approximate balance for breed, gender and sire combinations between three groups. The said allocation technique also imposes constraints on inter-group variations in mean, as well as standard deviation, of age, weaning weight and average daily growth rate to weaning. With spring calving, these calves typically enter the NWFP in October at six months of age and are housed until the following April while being fed silage produced from their respective farmlets. Following winter housing, cattle are turned out to pasture and rotated around seven fields that constitute each farmlet. The animals are maintained until they reach target weights of ∼555 kg for heifers and ∼620 kg for steers (typically around October) with sufficient muscle coverage (conformation) and fat cover (fat class) for the UK market.

For the present experiment, one field from each farmlet was selected based on similarities with regards to field size and animal movements. The three fields used were: Orchard Dean South, Higher Wyke Moor and Poor Field (https://nwfp.rothamsted.ac.uk). Historically, all three farmlets at the NWFP were uniformly managed as permanent pasture until 2013 to test their inter-comparability (Takahashi et al., 2018). From 2013 onward, two of the farmlets were gradually converted over three grazing seasons to different sward types representing two common alternatives to permanent pasture in temperate regions (as described above). Amongst the three fields used in the present study, Orchard Dean was kept under PP, Higher Wyke Moor was converted to WC in July 2013 and Poor Field was converted to HS in July 2014. Under the NWFP’s farm management plan, these fields were exclusively allocated to cattle grazing (as part of a multi-field rotation) during the experiment period and, to protect the field equipment, the experimental area was secured using electric fencing when the rest of the field was occupied by animals.

**Fig. 1:**
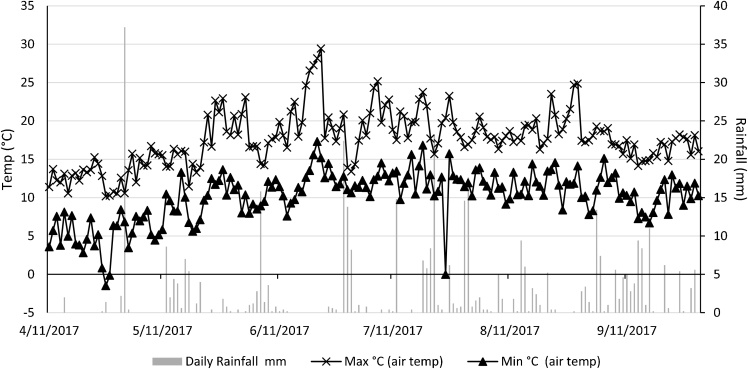
Mean daily temperature and rainfall from the North Wyke meteorological station during the sampling campaign. Treatment application occurred on 06/06/2017.

Soils on all three fields are typical non-calcareous pelosol of the Halstow and Hallsworth series according to the British soil classification (Mückenhausen, 1981), whereas FAO (2015) and USDA (1999) classification systems define them as Stagni-vertic cambisol and aeric haplaquept, respectively. Table 1 shows the soil physicochemical characteristic of the three fields. The climate in the region is temperate maritime (Mückenhausen, 1981) typical of South West England. Rainfall is generally highest in December (130 mm) while temperature tends to peak in July (max. = 19.9 °C; min. = 12.0 °C) and August (max. = 19.8 °C; min. = 12.1 °C). Lowest values are observed during June for rainfall (55.9 mm), and February (max. = 7.7 °C; min. = 2.1 °C) for temperature. Daily rainfall and temperature data were sourced from the Met Office weather station situated on-site. Rainfall events and temperatures recorded during the sampling campaign are depicted in Fig. 1.

**Table 1:** Soil characteristics of each farmlet. These data are based on a North Wyke Farm Platform soil survey which was carried out in July 2016.

Farmlet	Permanent pasture (PP)	White clover mix (WC)	High sugar grass (HS)
Field name	Orchard Dean South	Higher Wyke Moor	Poor Field
Soil type	Clay	Clay	Clay
pH	5.64	5.47	5.74
Total C (%w w^−1^)	5.95	3.87	3.88
Total N (%w w^−1^)	0.62	0.40	0.41
C:N ratio	9.60	9.68	9.46
Bulk density (g cm^−3^)	0.88	0.98	1.08

N_2_O measurements were conducted between 11/04/2017 and 27/09/2017 (details below). In comparison to the 30-year mean, rainfall was higher throughout this period, with the highest monthly total (105 mm) occurring in July (Fig. 1). Total rainfall in the 30 days following treatment application (06/06/2017) was 55 mm, with the distribution skewed towards the end of the period after a comparatively dry period. From treatment application to the conclusion of the trial, 296 mm of precipitation was recorded in total. In contrast to rainfall, air temperature followed a similar pattern to the 30-year mean, although minimum monthly means were slightly higher during the experiment. The highest maximum daily temperature recorded was 29 °C (22/06/2017), whereas the lowest minimum was −2 °C (26/04/2017). On the day of treatment application, temperatures were 14.4 °C (maximum) and 8.9 °C (minimum). Mean maximum and minimum temperatures for 60 days following treatment application were 19.8 °C and 12.1 °C, respectively.

### 2.2 Experimental design

On each of the three fields, three experimental blocks (15 m × 1.5 m) were established 50 m away from each other in an equilaterally triangular pattern. As stated above, these blocks were fenced off while animals were grazing on the same field but otherwise managed similarly to the rest of the pasture. Each block was further divided into six plots (2.5 m × 1.5 m) laid along a contour and randomly assigned to either a treatment or a control (Supplementary Fig. S1). Treatments were defined as dung (D), cattle urine (CU) and synthetic urine (SU). SU was used as a standard treatment across the three fields to allow the direct (unconfounded) effect of pasture composition on N_2_O emissions. On PP and HS, two control plots received inorganic N in an identical manner as the treatment plots and the rest of the field (CON + N), while another received no N fertiliser (CON-N). As WC farmlet does not receive any inorganic N due to the N-fixation capacity of white clover, WC had three CON-N plots. Where relevant, inorganic N was applied in the form of ammonium nitrate three times during the grazing season at a rate of 40 kg N ha^−1^ per application (120 kg N ha^−1^ in total). The second CON + N plot in each block (or the third CON-N plot in the case of WC) was initially intended for a staggered application of cattle urine later in the season; however, a second round of urine collection proved infeasible due to practical constraints, and the decision was made to manage this “spare” plot as an additional control, rather than abandoning it, to maximise statistical power. Within each plot, two static chambers (40 cm × 40 cm) were symmetrically installed alongside each other and a 1 m^2^ area was set aside for soil sampling and yield assessment.

Urine on both CU and SU plots was applied at a rate of 5 l m^−2^ (de Klein et al., 2014), while dung on D plots was applied at a rate of 20 kg m^−2^ (Sugimoto and Ball, 1989). These values represent approximate rates typically returned during a single deposition event (Cardenas et al., 2016). Synthetic urine was prepared according to a standard recipe developed by Kool et al. (2006) and described elsewhere (Cardenas et al., 2016). Urine from cattle was collected from cattle grazing in each farmlet after segregating steers and heifers. Urine from steers was obtained in the cattle handling facility via a bucket with an extended handle during natural urination events. Meanwhile, heifers were put through a cattle crush and, one at a time, encouraged to urinate via vulva stimulation. Cattle urine was then frozen at −20 °C in sealed containers until three days before application, when it was gradually defrosted in a fridge at 4 °C and then mixed together into bulked barrels for PP, WC and HS separately. For dung, the freshest deposits on pastures were subjectively identified based on the degree of crust development typically associated with decomposition, collected with a ladle and then placed into a barrel. Following on-field collection, samples were homogenised and refrigerated in sealed containers at 4 °C until the day of application. Subsamples of dung were analysed for total carbon (TC) and total nitrogen (TN) contents using Carlo Erba NA 1500 analyser (CE Instruments Ltd, Wigan UK) after ∼15 mg ground oven-dried material was weighed into a foil capsule. Meanwhile, urine subsamples were diluted 50-folds (0.5 mL urine was made up to 25 mL with ultra-pure milli-Q water) and then analysed for TN and TC contents using a Shimadzu Total Organic Carbon Analyser TOC-L Series. Urine pH was determined from undiluted urine using a Jenway 3320 pH meter.

Treatments were applied on 06/06/2017. Cattle urine and synthetic urine were applied to the area inside chambers (0.16 m^2^ each, details below) and the surrounding area using watering cans with perforated spray heads on the CU and SU plots, respectively. Dung was applied evenly to the D plots, both inside and outside chambers, at the aforementioned rate. The resulting N application rates for all treatments are shown in Table 2.

**Table 2:** Urine and dung composition and standard error (SE) for each farmlet.

	Permanent pasture (PP)		White clover mix (WC)		High sugar grass (HS)	
	Urine	Dung	Urine	Dung	Urine	Dung
	mg L^−1^	mg kg DM^−1^	mg L^−1^	mg kg DM^−1^	mg L^−1^	mg kg DM^−1^
Total N concentration	3311 (32)	33.6 (0.2)	1958 (34)	31.7 (0.3)	1733 (20)	34.6 (0.4)
Total C concentration	7294 (74)	427 (3)	6501 (136)	409 (3)	4522 (108)	425 (4)
Total P concentration	n.d.	10,087 (89)	n.d.	9476 (54)	n.d.	10,118 (82)
% DM	n.d.	9.87 (0.05)	n.d.	8.8 (0.05)	n.d.	11.44 (0.05)
pH	8.64 (0.01)	n.d.	8.46 (0.01)	n.d.	8.63 (0.01)	n.d.

n.d. = not determined.

Following the NWFP’s management practices, inorganic N fertiliser was applied to PP and HS plots (except for CON-N) on 10/04/2017, 08/05/2017 and 05/06/2017. To ensure chambers received the correct amount of fertiliser, they were closed during application by tractor and spreader, and subsequently received the same fertiliser at an equal rate by hand (40 kg N ha^−1^ or 1.85 g N chamber^−1^ per application event). CON-N plots were completely covered with silage tarpaulin and did not receive any form of supplementary N.

### 2.3 Sampling strategies and measurements

#### 2.3.1 N_2_O emissions

Gas sampling and analyses were carried out according to an established experimental protocol (Chadwick et al., 2014). As described earlier, each plot had duplicated chambers, each 40 cm (length) × 40 cm (width) × 25 cm (height) in dimension and tightly inserted to ∼5 cm depth in the soil. Sampling commenced on 11/04/2017 (56 days before treatment application and one day prior the first fertiliser application) and concluded on 27/09/2017 (113 days after treatment application) when N_2_O emissions had largely returned to pre-treatment levels.

After each inorganic N application, N_2_O fluxes were measured three times per week for two weeks, after which the frequency was reduced to twice-weekly. This pattern continued until the application of urine and dung. From this point, gas samples were collected three times a week for the first two weeks then twice a week for a further 10 weeks to maximise the accuracy of recorded N_2_O emissions from treatment application. Subsequently, sampling frequency was reduced to once fortnightly until termination of the sampling campaign. Overall, this resulted in 43 individual sampling days over a 169-day period. Five ground-level atmospheric samples were collected at approximately 11:00 (T0) on each sampling day. Chamber lids were then placed sequentially across all plots and, after 40 min from this moment, a gas sample was collected directly from each closed chamber (T40). Gas fluxes were calculated based on the linear increase in gas concentration inside the chamber from T0 to T40 (Smith and Dobbie, 2001). Chamber heights were measured inside in 4 points and the average value was used to calculate the emissions. In addition, one chamber per block (always under SU treatment) was designated for a linearity check of gas accumulation in the headspace (Chadwick et al., 2014), where in addition to T0 and T40, samples were also collected at 20 min (T20) and 60 min (T60) following chamber closure. In total, 125, 129 and 122 chambers were tested for linearity on PP, WC and HS, respectively, with the discrepancies resulting from sampling errors or damaged chambers. Under these tests, 88 % of WC chambers as well as 83 % of PP and HS chambers were shown to be linear, defined by R^2^ greater than 0.5, indicating that most emissions were captured by the adopted method. Cumulative emissions were derived using trapezoidal integration (Cardenas et al., 2010). Soil surface temperature was measured at every gas sampling event using a portable thermometer (Fisher Scientific, UK) to adjust gas concentrations to appropriate temperatures during calculation of fluxes.

EFs were calculated according to the definition by Venterea et al. (2015) as % N input (as urine, dung or fertiliser) lost as N_2_O-N. Specifically, cumulative N_2_O-N emissions from CON-N plots were subtracted from the cumulative emissions in each treatment, and this difference was divided by the total amount of N applied to the treatment plots. EFs were first calculated on a per chamber basis and then averaged across chambers and blocks.

#### 2.3.2 Soil

On each gas sampling day, soil moisture was measured by the gravimetric method using samples dried at 105 °C. Representative soil samples were taken at 0−10 cm depth from CON-N and D plots from the areas allocated to soil sample collection. Selection of CON-N plots was motivated by the greater comparability of results between WC and PP/HS, as the latter two systems received inorganic N on all but these plots, while separate sampling from D plots was necessitated due to the unique property of this treatment to physically conceal soil surface. Due to the size of the plots, which were restricted because of the need to provide sufficient pasture for grazing cattle, soil samples for moisture determination were not taken for all treatments. However, multiple spot sampling resulted in no statistically significant difference (*p* = 0.66) amongst all but D plots within a single block. Soil bulk density (BD, g cm^−3^) was measured after treatment application in each plot. Metal rings were used to sample soil cores that were weighed after drying to determine the soil weight that occupied the ring volume. Water-filled pore space (WFPS, %) was subsequently calculated on CON-N and D plots using BD and gravimetric soil moisture. Soil samples were collected at three timepoints (02/06/2017, 02/08/2017 and 10/10/2017) across the sampling campaign and analysed for % mass of TC and TN after being freeze-dried, ground and weighed into a foil capsule. The amount of subsamples used for this analysis corresponded to the amount required to detect TN and varied between 40 and 100 μg.

#### 2.3.3 Forage

Herbage samples were collected from a designated 50 cm × 50 cm area (details above) three times (23/05/17, 10/08/17 and 10/10/17) during the campaign, once before and twice after treatment application. Dry matter (DM) yield was determined after oven-drying material at 85 °C for 24 h. Forage samples were freeze-dried and WSC were extracted by adding 20 mL of milli-Q H_2_O to 200 mg of a given sample and shaking for an hour at room temperature. Extracts were filtered and stored at −20 °C until being analysed using an Agilent 1260 Infinity HPLC system consisting of a quaternary pump, degasser, autosampler and a heated column running at 40 °C (Agilent Hi-PLEX H column 300 × 7.7 mm) using 0.1 % trifluoro acetic acid (Fisher scientific) made with Milli-Q H_2_O as mobile phase. The instrument was controlled and analysed using Agilent Openlab software, with the ELSD settings of evaporator = 90 °C, nebuliser = 50 °C and Flowrate = 1.1 SLM. Herbage TN concentration was analysed in the same manner as soil, with the exception that a larger amount of sample (2−3 mg) was weighed into a capsule to ensure accurate detection.

#### 2.3.4 DNA extraction and quantification

Soil samples for DNA extraction were collected three times (02/06/2017, 30/06/2017 and 10/10/2017) using a stainless-steel cork borer (1 cm × 10 cm), which was manually inserted into the different plots, with eight cores bulked into four samples per treatment. These samples were snap frozen in liquid nitrogen immediately following collection2.3.2 and stored at −80 °C. The samples were subsequently prepared for DNA extraction by homogenising four replicate samples in a chilled blender. DNA was extracted from 0.5 g soil using a PowerSoil DNA extraction kit (Mo Bio Laboratories, Carlsbad, CA) following the manufacturer’s instructions, except for using a FastPrep-24 5 G Homogenizer (MP Biomedicals Santa Ana, CA, USA) at step five. DNA was quantified using a Qubit 3 fluorometer and diluted to 5 ng μl^−1^. The abundance of bacteria (16SB), archaea (16SA) and fungi (ITS) was estimated by quantitative PCR (qPCR) of the 16S rRNA and of the internal transcribed space gene as molecular markers; the size of the nitrifier community was quantified after amplification of the *amoA* gene from archaea (*amoA* AOA) and bacteria (*amoA* AOB), and that of denitrifiers by qPCR of the *nirK*, *nirS, nosZ*I and *nosZ*II genes. Quantitative amplifications were performed in accordance with the MIQE guidelines (Bustin et al., 2009). The qPCR was carried out with Quantifast SYBR Green PCR Kit in a Biorad CFX384 Touch real time PCR detection system. qPCR reaction mixtures contained 5 μL of Quantifast SYBR Green PCR Kit, 0.1 μL of each primer (100 μM), 2 μL of H_2_O and 4 μL of DNA (5 ng μl^−1^). The primers used for amplification of each gene are displayed in Supplementary Table S1, along with the cycling conditions. To provide absolute quantifications of the target organisms, standard curves were generated with serial tenfold dilutions (10^−1^–10^−8^) of linearized standard plasmids carrying inserts of the target genes. The standards used for the quantification of targeted genes were previously constructed (Clark et al., 2012). Positive controls containing REfDNA (mix of extracted DNA from arable soil (25 %), grassland (25 %) and wilderness (50 %) soils at 5 ng μl^−1^) were also run alongside three negative controls containing no DNA. The calibration curves resulted in R^2^ > 0.99 in all assays. The efficiency of PCR amplification for all target genes was between 90 and 100 %.

#### 2.3.5 Statistical analysis

Statistical analysis was conducted using GenStat V18 (www.vsni.co.uk). Cumulative emissions were log-transformed to account for skewed residual distributions and subsequently analysed using linear mixed models (REML) with a fixed model structure of farmlet × treatment and a random model structure of block/plot/chamber. Microbial abundance was also log-transformed and analysed using REML, however the relevant timepoints were accounted for in both the fixed and random models. REML was chosen over analysis of variance (ANOVA) due to the uneven design of the experiment, resulting from the fact that there were three CON-N plots on WC and one on PP and HS (Section 2.2). Effects of treatments and farmlets as well as their interactions were examined. REML was also used to analyse qPCR results to test for differences in abundances of N cycling genes. The fixed structure of the model included an additional factor which identified which gene each observation related to. This addition helped to identify the dominant gene(s) for each farmlet/treatment/timepoint combination and determine if they changed before and after treatment. The factors studied were farmlet, treatment, timepoint, gene and their two-way and three-way interactions. Backward selection was used to simplify the microbial model; this led to all terms involving an interaction between farmlet and treatment being dropped out. In addition, differences in WFPS across farmlets and between CON-N and D plots were tested using two-way ANOVA. To investigate the relationship between WFPS and N_2_O emissions, a simple linear regression was carried out. Finally, REML was used to determine if there were any differences in soil and forage quality.

## 3 Results

### 3.1 Urine and dung properties under different swards

The composition of urine and dung collected is shown in Table 2. The TN concentration in cattle urine for all three farmlets was lower than in the synthetic urine (10,037 ± 100 mg N L^−1^). The highest N concentration in urine obtained from cattle was on the PP farmlet (3311 ± 32 mg N L^−1^) whereas animals on the HS farmlet showed the lowest urine N concentration (1733 ± 20 mg N L^−1^). Dung composition was less variable between the farmlets. HS showed the highest dung N concentration (34.6 ± 0.4 mg N kg DM^−1^) whereas the lowest was found in WC (31.7 ± 0.3 mg N kg DM^−1^).

As urine and dung N concentrations differed between farmlets, N amounts applied differed between the three systems, with cattle dung always providing higher N concentration than cattle urine; this is despite the same volume of urine and dung being applied to each farmlet to simulate a single deposition event. In the dung treatments, HS had the highest N application rate (791 kg N ha^−1^), followed by PP (664 kg N ha^−1^) and WC (559 kg N ha^−1^) (Table 3). Amongst cattle urine treatments, the highest N application occurred on PP (286 kg N ha^−1^), followed by HS (207 kg N ha ^−1^) and WC (98 kg N ha^−1^). Thus, total N applied to the plot, including fertiliser N where applicable, was highest in the dung treatments, with HS receiving the highest rate (911 kg N ha^−1^) followed by PP (784 kg N ha^−1^) and WC (559 kg N ha^−1^).

**Table 3:** Summary of N inputs to each treatment. All values reported as kg N ha^−1^. Treatment applications occurred on 06/06/2017, while fertiliser N applications (at 40 kg N ha^−1^) were carried out on 10/04/2017, 08/05/2017 and 05/06/2017.

Farmlet	D			SU			CU			CON + N	
	Fert-N	D-N	Total N	Fert-N	SU-N	Total N	Fert-N	CU-N	Total N	Fert-N	Total N
PP	120	664	784	120	502	622	120	166	286	120	120
WC	–	559	559	–	502	502	–	98	98	–	–
HS	120	791	911	120	502	622	120	87	207	120	120

PP = permanent pasture; WC = white clover/high sugar grass mixed sward; HS = high sugar grass monoculture.

D = dung; SU = synthetic urine; CU = cattle urine; CON + N = control with synthetic nitrogen fertiliser.

### 3.2 N_2_O emissions under different treatments and swards

#### 3.2.1 Daily fluxes

**Fig. 2:**
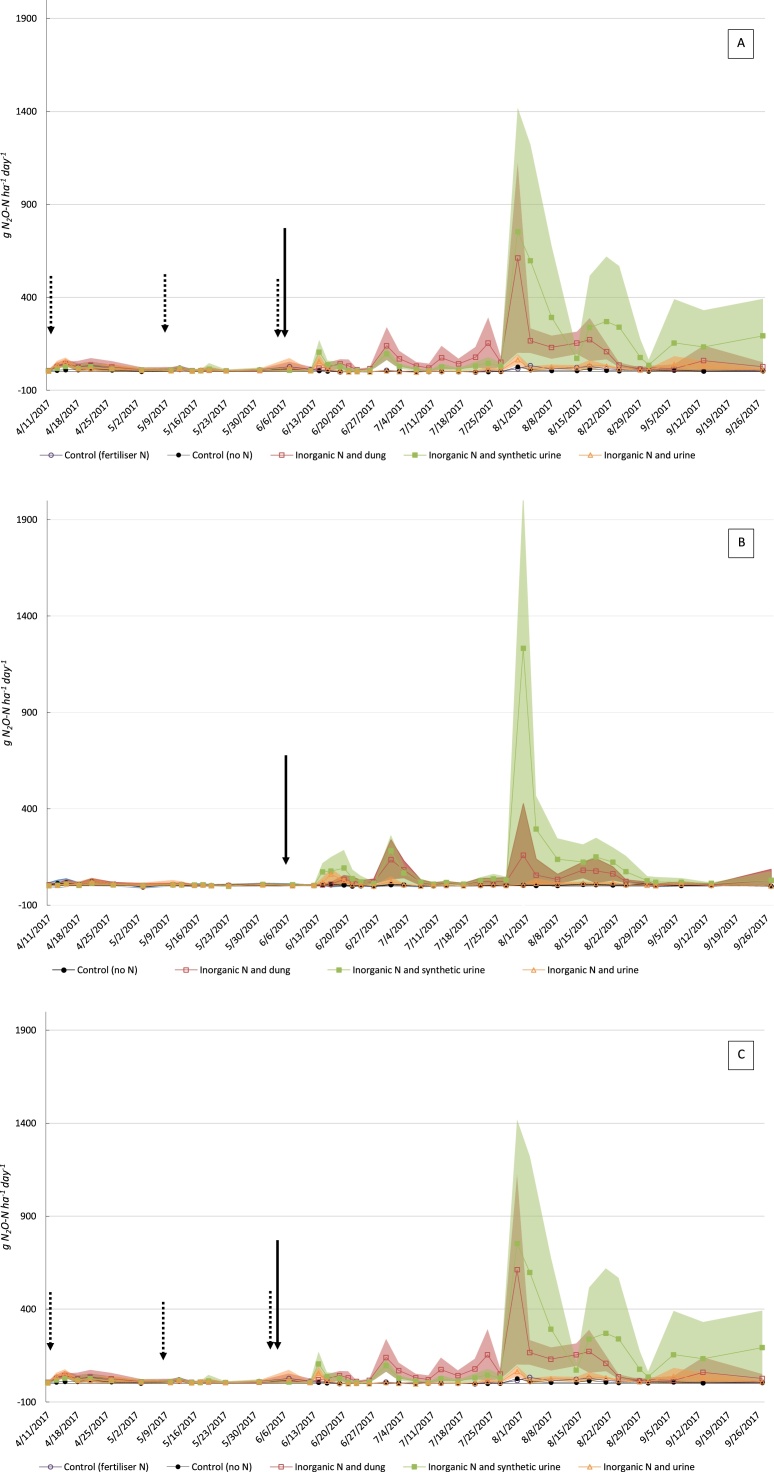
Daily N_2_O-N fluxes for each farmlet. A: permanent pasture (PP); B: white clover/high sugar grass mix (WC); C: High sugar grass monoculture (HS). Treatments were applied on 6th June (see large black arrow). Smaller dotted arrows denote inorganic fertiliser application (40 kg N/ha/application). Shaded areas signify standard deviation.

Across all farmlets, the largest N_2_O fluxes were found on SU, with maximum daily peaks of 459, 1233 and 754 g N_2_O-N ha^−1^ d^−1^ on PP, WC and HS, respectively (Fig. 2). Following treatment application on 06/06/2017, there were modest spikes on D, CU and SU plots; however, the largest fluxes did not occur until the end of July, when temperature and rainfall both increased substantially (Fig. 1). After SU, D produced the second largest daily fluxes, with HS generating a notably high peak (612 g N_2_O-N ha^−1^ d^−1^), followed by PP (236 g N_2_O-N ha^−1^ d^−1^) and WC (159 g N_2_O-N ha^−1^ d^−1^), all recorded 55 days after treatment application (31/07/2017). The CU treatment produced considerably lower N_2_O emissions in comparison to SU across farmlets and time; for instance, the maximum daily fluxes for CU were 115 and 65 g N_2_O-N ha^−1^ d^−1^ recorded 55 days after treatment application for PP and HS, respectively, and 61 g N_2_O-N ha^−1^ d^−1^ for WC recorded 10 days after treatment application. Both controls exhibited comparatively lower fluxes, with CON + N generating larger peaks compared to CON-N on PP and HS. Fluxes on WC control plots were comparatively low, a result likely explained by the absence of inorganic fertiliser applications.

#### 3.2.2 Cumulative emissions

Total emissions across the experiment are summarised in Table 4. The results of REML analysis revealed that these values were significantly different amongst treatments (*p* < 0.001) and farmlets (*p* = 0.022). However, no significant interaction between treatment × farmlet was observed (*p* = 0.411). SU had the highest emissions across all farmlets, an expected result given the larger amount of N present in the synthetic urine compared to cattle urine (Section 3.2). Cumulative N_2_O emissions were found to rise exponentially as total N input to the plot increases (Supplementary Fig. S2). In addition, a significant farmlet effect on cumulative N_2_O emissions from the CU and D treatment was observed, with HS showing the highest total emissions both for CU (2243.9 g N_2_O-N ha^−1^) and D (9638.3 g N_2_O-N ha^−1^) followed by PP (1644.4 g N_2_O-N ha^−1^ for CU and 5152.3 g N_2_O-N ha^−1^ for D) and WC (1083.9 g N_2_O-N ha^−1^ for CU and 3303.7 g N_2_O-N ha^−1^ for D). CON + N generally produced higher emissions than CON-N. The EFs for CON + N treatment were 1.38 and 0.73 % for PP and HS, respectively (Table 5). The proportion of total N lost as N_2_O on the SU treatment was higher than that calculated for CU on all farmlets. The EFs for SU were between 1.12 (PP) and 2.43 % (HS), whereas for CU values ranged between 0.55 (PP) and 0.76 % (HS). For D treatment, the EFs ranged between 0.60 (PP) and 0.98 % (HS).

**Table 4:** Cumulative N_2_O-N emissions (as g N_2_O-N ha^−1^).

Treatment	CON + N		CON-N		CU		D		SU	
	PM	(95 % CI)	PM	(95 % CI)	PM	(95 % CI)	PM	(95 % CI)	PM	(95 % CI)
Total N_2_O PP	1614.36	(1039.9–2506.16)	375.84	(202.66–696.99)	1644.37	(886.7–3049.47)	5152.29	(2778.28–9554.85)	7014.55	(3782.47–13008.4)
Total N_2_O WC	*	*	522.40	(362.66–752.48)	1083.93	(584.49–2010.13)	3303.70	(1781.46–6126.66)	9794.90	(5281.73–18164.52)
Total N_2_O HS	1406.05	(905.72–2182.77)	703.07	(379.12–1303.84)	2243.88	(1209.97–4161.25)	9638.29	(5197.28–17874.09)	13273.94	(7157.74–24616.37)

* This treatment did not receive fertiliser N; PM = predicted mean; CI = 95 % confidence interval predicted by REML analysis.

PP = permanent pasture; WC = white clover/high sugar grass mixed sward; HS = high sugar grass monoculture.

CON + N = control with synthetic nitrogen fertiliser; CON-N = control with no amendments; CU = cattle urine; D = dung; SU = synthetic urine.

**Table 5:** Emission factors (defined as proportion of N lost as N_2_O-N relative to N-inputs) for each treatment and farmlet.

Treatment	CON + N	CU	D	SU
EF N_2_O PP, %	1.38	0.55	0.60	1.12
EF N_2_O WC, %	*	0.69	0.64	2.11
EF N_2_O HS, %	0.73	0.76	0.98	2.43

* This treatment did not receive fertiliser N.

PP = permanent pasture; WC = white clover/high sugar grass mixed sward; HS = high sugar grass monoculture.

CON + N = control with synthetic nitrogen fertiliser; CU = cattle urine; D = dung; SU = synthetic urine.

### 3.3 Soil properties under different treatments and swards

**Fig. 3:**
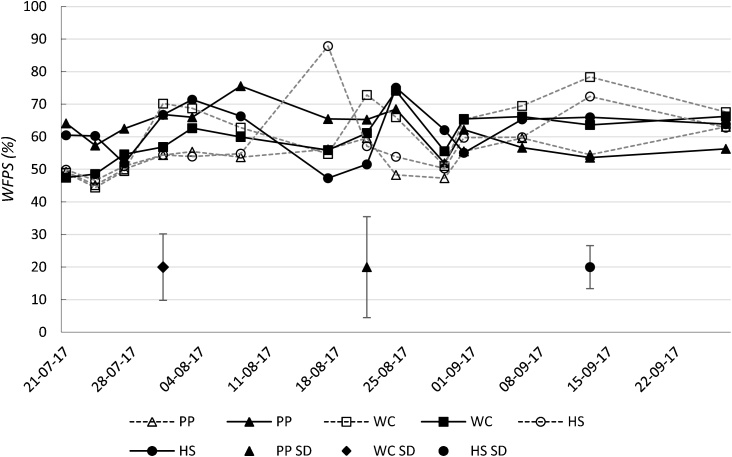
Water filled pore space (WFPS) (as % fresh soil) from designated moisture controls and dung plots on each farmlet. Results presented are post-treatment. Error bars represent the average standard deviation (SD) across all treatments. PP = permanent pasture; WC = white clover/high sugar grass mixed sward; HS = high sugar grass monoculture.

Across all farmlets throughout the entire sampling campaign, soil moisture (measured as WFPS) was in the range of 44 % to 88 % (Fig. 3). The mean values were 58.0 %, 61.0 % and 59.9 % for PP, WC and HS, respectively. On the two treatments where moisture was measured (CON-N and D), there were significant effects from farmlet (*p* < 0.001) and the farmlet × treatment interaction (*p* = 0.01); the treatment effect was relatively weaker (*p* = 0.09). Although a positive association was identified between WFPS and N_2_O emissions, this was not found to be significant (*p* = 0.25). Regression analysis predicted that, WFPS being equal, HS had significantly higher emissions than PP (*p* = 0.044) and WC (*p* = 0.031).

Prior to treatment application, there were no significant differences for soil TC across farmlets (*p* = 0.167) nor treatments (*p* = 0.917). The same was true for TN across farmlets (*p* = 0.074) and treatments (*p* = 0.810). Throughout the duration of the experiment, however, TN in soil was found to differ across farmlets (*p* < 0.001), timepoints (*p* < 0.001), and, although not statistically significant at the 5 % level, treatments (*p* = 0.061). On average across time, PP consistently showed a higher soil-N concentration than WC and HS; on average across farmlets, the highest N concentration was recorded directly after treatment application. D plots had the highest TN values within each farmlet with 0.711, 0.499 and 0.467 % being recorded on PP, WC and HS, respectively (Table 6). The lowest values tended to be on the CON plots, except in the case of HS, where SU had lowest soil TN (0.397 %) following treatment application.

**Table 6:** Predicted means of soil total N concentration (%) in the three pasture systems from REML at the three different time points (time point 1 = 4 days before treatment application; time point 2 = 57 days after treatment application; time point 3 = 126 days after treatment application).

Treatment	Time point	PP	(95 % CI)	WC	(95 % CI)	HS	(95 % CI)
D	1	0.599	(0.54−0.659)	0.445	(0.386−0.504)	0.423	(0.364−0.483)
	2	0.711	(0.651−0.77)	0.499	(0.439−0.558)	0.463	(0.404−0.523)
	3	0.617	(0.557−0.676)	0.412	(0.352−0.471)	0.467	(0.408−0.527)
SU	1	0.644	(0.585−0.704)	0.449	(0.39−0.508)	0.418	(0.359−0.478)
	2	0.672	(0.613−0.731)	0.474	(0.414−0.533)	0.397	(0.338−0.456)
	3	0.579	(0.52−0.638)	0.419	(0.36−0.478)	0.432	(0.373−0.491)
CU	1	0.644	(0.584−0.703)	0.429	(0.37−0.489)	0.429	(0.37−0.488)
	2	0.655	(0.595−0.714)	0.424	(0.365−0.484)	0.432	(0.373−0.491)
	3	0.560	(0.5−0.619)	0.398	(0.339−0.458)	0.407	(0.347−0.466)
CON + N	1	0.632	(0.573−0.692)	–	–	0.428	(0.369−0.487)
	2	0.683	(0.624−0.743)	–	–	0.436	(0.376−0.495)
	3	0.510	(0.451−0.569)	–	–	0.424	(0.364−0.483)
CON-N	1	0.592	(0.532−0.651)	0.444	(0.396−0.492)	0.455	(0.396−0.515)
	2	0.636	(0.577−0.695)	0.450	(0.402−0.498)	0.432	(0.373−0.492)
	3	0.555	(0.496−0.615)	0.394	(0.346−0.442)	0.405	(0.345−0.464)

Note: 95 % CI = confidence intervals of predicted values from REML analysis.

PP = permanent pasture; WC = white clover/high sugar grass mixed sward; HS = high sugar grass monoculture.

D = dung; SU = synthetic urine; CU = cattle urine; CON + N = control with synthetic nitrogen fertiliser; CON-N = control with no amendments.

### 3.4 Forage properties under different treatments and swards

Before treatment application, average herbage N concentrations for all treatments were higher in the PP pasture (2.93 %) than in the other two pastures (2.13 and 2.49 % in WC and HS, respectively). After treatments were applied, N concentration in herbage only increased for WC (to 2.65 %), while the remaining farmlets observed a slight decrease (to 2.51 and 2.22 % in PP and HS, respectively). The predicted means of % N in herbage from REML considering “cut” (i.e. three timepoints) as an effect are shown in Table 7. REML analysis demonstrated that both treatment and farmlet had significant effects (p < 0.001 and p = 0.002, respectively). For the two-way interactions, all were significant at *p* < 0.001 for farmlet × cut and *p* = 0.002 for farmlet × treatment and treatment × cut.

**Table 7:** Predicted means of percentages of water-soluble carbohydrate (WSC), and N concentration and DM yield in the three pasture systems from REML. Percentages reported in relation to dry matter (DM).

Treatment		PP	(95 % CI)	WC	(95 % CI)	HS	(95 % CI)
D	WSC	12.18	(9.23–15.13)	14.54	(11.76–17.32)	15.27	(12.49–18.05)
	N	3.00	(2.68–3.31)	2.42	(2.2–2.64)	2.53	(2.29–2.78)
	DM	57.97	(39.94–76)	73.92	(57.01–90.83)	73.26	(56.35–90.17)

SU	WSC	11.21	(8.43–13.99)	12.77	(9.99–15.55)	13.69	(10.91–16.47)
	N	2.90	(2.68–3.12)	2.56	(2.35–2.78)	3.06	(2.75–3.38)
	DM	86.80	(69.89–103.71)	87.69	(70.78–104.6)	99.67	(82.76–116.58)

U	WSC	10.72	(7.94–13.5)	15.23	(12.45–18.01)	16.49	(13.71–19.27)
	N	2.82	(2.6–3.04)	2.30	(2.08–2.51)	2.19	(1.94–2.44)
	DM	79.65	(62.74–96.56)	76.16	(59.25–93.07)	112.94	(96.03–129.85)

CON + N	WSC	12.72	(9.94–15.5)	–	–	16.60	(13.82–19.38)
	N	2.74	(2.53–2.96)	–	–	2.18	(1.86–2.49)
	DM	79.11	(62.2–96.02)	–	–	73.42	(56.51–90.33)

CON-N	WSC	14.86	(12.08–17.64)	16.47	(14.35–18.59)	20.09	(17.31–22.87)
	N	2.16	(1.94–2.37)	2.29	(2.11–2.46)	1.81	(1.48–2.13)
	DM	71.48	(54.57–88.39)	63.07	(50.82–75.32)	59.56	(42.65–76.47)

Note: 95 % CI = confidence intervals of predicted values from REML analysis.

PP = permanent pasture; WC = white clover/high sugar grass mixed sward; HS = high sugar grass monoculture.

D = dung; SU = synthetic urine; CU = cattle urine; CON + N = control with synthetic nitrogen fertiliser; CON-N = control with no amendments.

Table 7 also displays predicted means for DM yield in herbage from REML, again considering “cut” (equal in meaning to “time-point”) as an effect. The results from REML showed that effects from both treatment and cut were significant (*p* < 0.001), whereas farmlet did not demonstrate a significant effect (*p* = 0.225). Treatment had a significant effect on total DM yield across all cuts (*p* < 0.001) whilst farmlet effect was not significant (*p* = 0.263). As expected, HS had significantly (*p* = 0.028) greater WSC concentrations than the other two pastures regardless of treatment. Specifically, the mean WSC content in the sward was 12.3, 14.8 and 16.4 % on PP, WC and HS, respectively.

### 3.5 Soil microbial properties under different treatments and swards

**Fig. 4:**
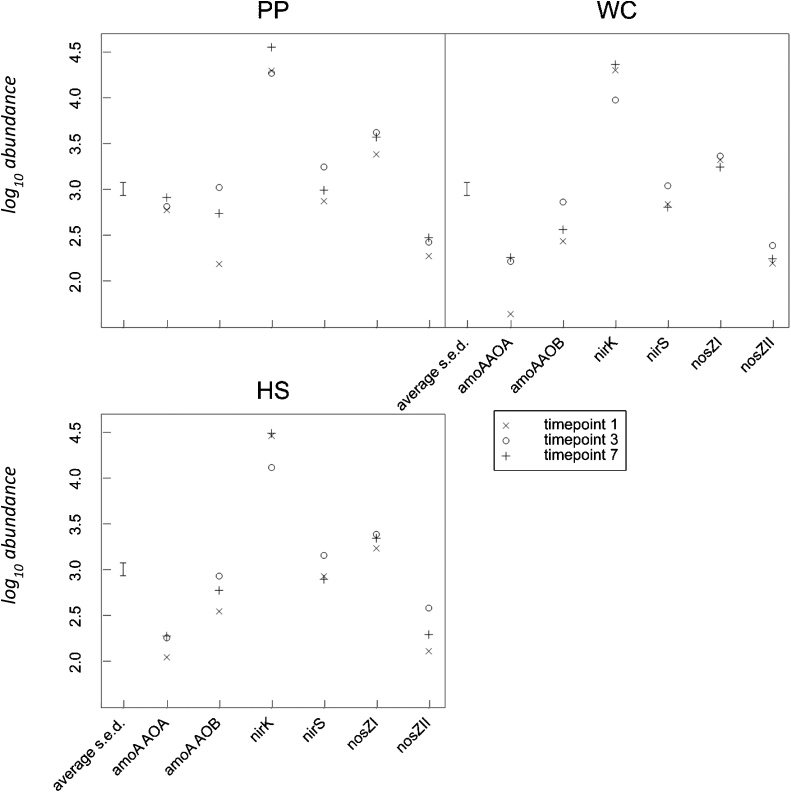
Total abundance of the amoA AOB, amoA AOA, nirK, nirS, nosZI and nosZII for all treatments within farmlets. Time point 1 = 4 days before treatment application (02/06/17); time point 3 = 24 days after treatment application (30/06/17); time point 7 = 126 days after treatment application (10/10/17). Values are expressed as log gene copy number × g^−1^ dry soil. PP = permanent pasture; WC = white clover/high sugar grass mixed sward; HS = high sugar grass monoculture.

**Fig. 5:**
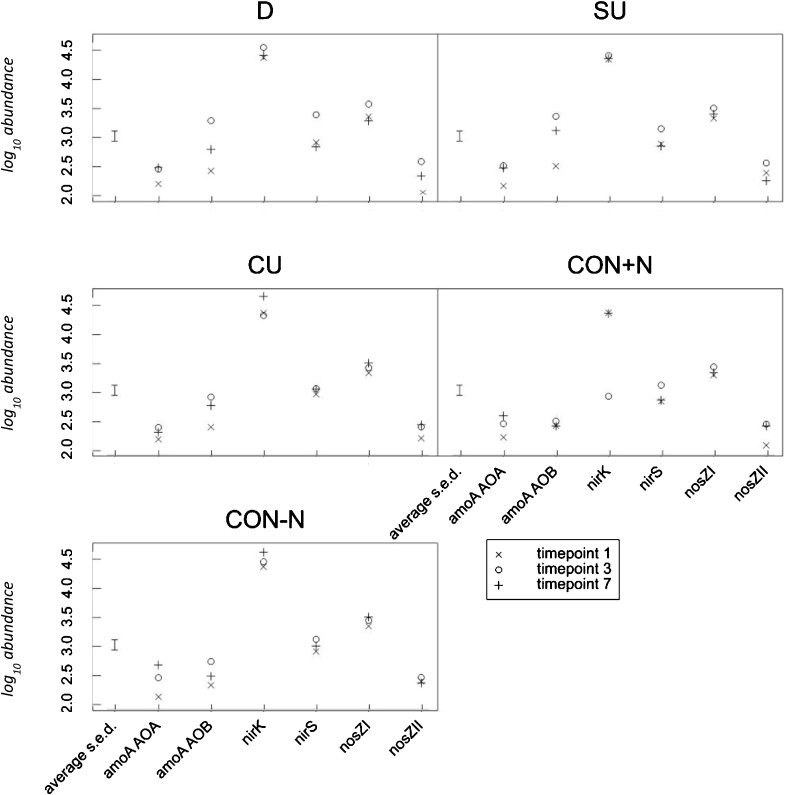
Total abundance of the amoA AOB, amoA AOA, nirK, nirS, nosZI and nosZII genes for all farmlets within treatments. Time point 1 = 4 days before treatment application; time point 3 = 24 days after treatment application; time point 7 = 126 days after treatment application. Values are expressed as log gene copy number × g^−1^ dry soil. PP = permanent pasture; WC = white clover/high sugar grass mixed sward; HS = high sugar grass monoculture. D = dung; SU = synthetic urine; CU = cattle urine; CON + N = control with synthetic nitrogen fertiliser; CON-N = control with no amendments.

Chronological changes in total abundance of genes: 16SB, 16SA, ITS, *amoA* AOA*, amoA* AOB, *nirK*, *nirS*, *nosZ*I and *nosZ*II for all pastures and all applied treatments are presented in Supplementary data. The results demonstrate that members of bacteria were significantly (*p* < 0.001) more abundant than those of fungi and archaea in all pastures and treatments. Farmlet, timepoint and gene were found to have significant (*p* < 0.001) effects, as was treatment (*p* = 0.033). All two-way interactions were also significant (*p* < 0.001) except for farmlet × timepoint (*p* = 0.833) and treatment × timepoint (*p* = 0.106). For the three-way interactions, treatment × timepoint × gene and farmlet × timepoint × gene generated significant effects (*p* < 0.001 and *p* = 0.007, respectively). Fig. 4 provides the farmlet × timepoint × gene interaction, whereas the treatment × timepoint × gene interaction is shown in Fig. 5. The comparison between farmlets showed a general tendency that treatment application produced an increase in total abundance for all genes except the *nirK* gene which showed a decrease post-treatment for all farmlets (Fig. 4). Regardless of treatment and timepoint, the *nirK* gene showed the highest total abundance followed by the *nosZ*I and *nirS* genes, then the *nosZ*II gene (Fig. 5). At timepoint 1 and 3, the total abundance of the *amoA* AOB gene was always larger than that of the *amoA* AOA gene for all treatments and farmlets, except for the PP whereas the total abundance of the *amoA* AOB was lower than *amoA* AOA at timepoint 1. The abundance of the *amoA* AOB gene increased significantly after the application of the urine and dung treatments in all farmlets. This change was not observed in CON + N or CON-N plots (Fig. 5).

## 4 Discussion

### 4.1 Sward effect on urine and dung properties

The present findings show that consumption of different types of forage resulted in statistically significant changes in urinary N concentration in cattle. For instance, the lowest herbage N concentration was found on WC, which subsequently resulted in lower urine and dung N compared to the PP diet. This aligns with previous studies showing the potential of legume-based forages to increase the NUE of livestock systems (Marley et al., 2007). In a related study utilising excreta from sheep fed different silage types, Cardenas et al. (2007) reported causal relationships between various silage properties and excreta compositions; for example, WSC content of silage was found to affect concentrations of soluble protein and phenolic compounds in the slurries. In the current study, the lowest urine N concentration was found on the HS system, which also contained the highest levels of WSC. Consequently, these results reiterate previous findings that pastures with a higher level of available WSC may result in an improvement to the N-to-energy balance in the rumen, resulting in an increase to NUE which ultimately leads to a reduction in urinary N concentration (Miller et al., 2002; Edwards et al., 2007). In the present study, however, HS was also found to have the highest N_2_O emissions. This suggests that N_2_O emissions are not solely controlled by the N-concentration in urine but also by other factors associated with an animals’ diet and pasture management (Dijkstra et al., 2013; Chirinda et al., 2019).

Regardless of pasture composition, urinary N concentration in the present study was considerably lower than commonly reported values (Gardiner et al., 2016; Chadwick et al., 2018). For example, urine used in a previous experiment on the same site — sourced from dairy cattle reared at Reading, UK — had N concentrations of 8.6 g N L^−1^, much higher than the beef cattle values reported herein due to typically protein-richer diets offered to animals in dairy systems (Cardenas et al., 2016). A comparison of urine N concentration both prior to freezing and following defrosting indicated little volatilisation occurred, suggesting that the low N-concentration of CU reported above is accurate and was not affected by losses due to volatilisation. Although several authors have used synthetic urine instead of cattle urine when studying urine patch emissions (Kool et al., 2006; de Klein et al., 2014; Ciganda et al., 2019), differences identified in the current study surrounding the composition of beef-cattle urine vs synthetic urine (produced based on dairy-cow urine composition) seems to indicate that the use of artificial urine is not an all-round solution when studying emissions from urine deposition. This is because a small set of recipes cannot represent the wide variation in urine composition observed both within and across farming systems due to the production system, sward type, breed, age and gender. This finding also highlights the importance of considering the entire farming system during the derivation of site-specific EFs, including the impact of cattle diets on emissions via urine and dung.

### 4.2 Treatment and sward effects on N_2_O emissions

Nitrous oxide fluxes are known to be heavily influenced by environmental factors such as soil moisture that modulate microbial activity and transport of gases in soils. The optimal level of WFPS for N_2_O production is in the range of 70–80 % depending on soil type (Butterbach-Bahl et al., 2013). As previously discussed, there was a comparatively dry period with low rainfall directly after treatment application; although there was 15.8 mm of rainfall on the night before treatment application, the following 21 days had minimal precipitation and the highest maximum daily temperatures recorded throughout the campaign. This led to relatively low soil moisture and likely explains the low N_2_O responses during the first few weeks following treatment application. On all three farmlets, however, smaller peaks were observed approximately three weeks after treatment application for D, SU and CU treatments. As CON + N did not show any notable peak, it would appear that the erstwhile fluxes were the result of nitrification of NH_4_^+^ produced from hydrolysed urea within urine and dung under aerobic soil conditions. This is thought to be due to nitrification being the dominant process for N_2_O formation under aerobic conditions (i.e. WFPS < 60 %) (Bateman and Baggs, 2005). When major increases (e.g. >1000 g N_2_O-N ha^−1^ day^−1^ on WC and HS) in N_2_O emissions occurred in August, soil moisture had increased following several rainfall events, generating anaerobic conditions which favour denitrification (Loick et al., 2017). The delay in these larger peaks was likely due to the dry conditions throughout the summer. The current results from SU and D plots, under which the amount of N applied was largely comparable, also reaffirm the earlier finding by Lessa et al. (2014) and Cardenas et al. (2016) that urine generally produces more N_2_O emissions than dung (Table 4). However, this conclusion did not extend to CU plots, which received considerably lower levels of N due to lower N-concentrations in the cattle urine collected at the study site.

Further contrasts can be drawn from earlier GHG measurements at a non-grazed grassland field nearby to the current study. For example, Cardenas et al. (2016) recorded cumulative N_2_O values of 13258, 11,059 and 2501 g N_2_O-N ha^−1^ on a spring application of SU, CU and D, respectively, to permanent pasture. Total N inputs on the urine treatments varied between 405 and 481 kg N ha^−1^, which is considerably higher than CU in the current study but lower than SU, possibly due to the absence of fertiliser N application in Cardenas et al. (2016). A comparison of N_2_O emissions between the two studies demonstrates that SU results were largely comparable, with PP, WC and HS recording lower, similar and higher emissions, respectively, relative to the average value reported by Cardenas et al. (2016). CU emissions, however, were notably lower in the present work, likely due to the lower total N applied. In a two-year (2010−11) monitoring study (without treatment application) on a nearby site largely comparable to PP field at the present experiment, Horrocks et al. (2015) recorded a daily maximum flux of under 50 g N_2_O-N ha^−1^ d^-1^ on 13/05/10 (20 days following ammonium nitrate application). This value is considerably lower than the maximum daily N_2_O-N flux recorded (155 g N_2_O-N ha^−1^ d^−1^) on PP CON + N, the most comparable treatment in the current study. As the overall N application rates were comparable between these plots, the differences in N_2_O emissions were likely driven by soil moisture (WFPS), which was generally lower in 2010 (Horrocks et al., 2015).

Under the recently refined IPCC guidelines (IPCC, 2019), default EFs for urine, dung and chemical fertiliser applications should be separately derived from campaigns where N_2_O emissions have been measured for at least 30 days. As the current study measured N_2_O emissions for a period of 169 days, the ratio between N_2_O-N lost and N applied can be considered as site-specific EFs. As Cardenas et al. (2016) found that most emissions following a treatment application of either urine or dung occurred within five months on the studied soil, the emissions attributable to treatments are also thought to be largely captured in the current experiment. For cattle urine, IPCC refined the default EF under wet climates to 0.77 % with a 95 % uncertainty range of 0.03–3.82 %, whereas for cattle dung, the recommended EF is 0.13 % (0.00−0.53 %). The present results are in line with IPCC default values for CU and SU but values recorded on D were higher. The higher EFs found on SU relative to CU treatments irrespective of the farmlet suggests that there might be components contained in beef cattle’s urine that acts as an inhibitor of N_2_O emissions. The concentration of these compounds can vary with different animal diets (Ciganda et al., 2019); however, these fluctuations cannot be accounted for when preparing a limited variety of synthetic urine. Therefore, these results again highlight the importance of using natural urine, wherever possible, to accurately reproduce the diet-deposition-emissions nexus at assessment of N_2_O emissions from urine patches. It should also be reiterated that EFs derived from the current experiment embed interactions between inorganic N and organic N, a common phenomenon in commercial farming that is rarely addressed at field trials. Notwithstanding, EFs estimated here correspond to a single weather pattern and should therefore be interpreted with caution, especially with regards to potential consequences of a typically wetter spring, as interannual patterns are likely to bear implications on N_2_O emissions (Ammann et al., 2020). For CU and D, the derived EFs should not be extrapolated to N input levels outside the current levels either, given the frequently nonlinear relationship between N input and N_2_O-N emissions (Cardenas et al., 2010 and Supplementary Fig. S2).

For inorganic N fertiliser, IPCC’s refined EF is 1.6 % (with a range of 1.3–1.9 %). N lost from fertiliser-only treatments (CON + N) aligned with these values in the case of PP, but HS values were much lower (0.73 %) and outside the 95 % range. Combined with elevated WSC levels of HS herbage samples, current findings suggest that high sugar grasses demonstrate efficiency at maintaining N with regards to plant-soil interactions. This is in line with previous findings that the benefits of N utilisation in WSC-rich crops are superior to protein-rich grasses (Lee et al., 2002; Miller et al., 2002). However, despite the lowest urine N concentration being found on the HS farmlet, this sward simultaneously produced the highest total emissions both from CU and D, ultimately resulting in the highest EFs. Moreover, a comparison of the EFs between PP SU and HS SU, both of which received the same amount of N, showed higher EFs for HS, suggesting an inefficiency at maintaining N supplied in the form of organic N. It is also possible that high sugar grasses contribute more C to soil structure through root exudates increasing the potential for N_2_O production. Overall, therefore, the reduction in urinary N realised in the HS farmlet did not result in a reduction of N_2_O emissions, highlighting that, at the study site, HS had a higher environmental impact than PP and WC in terms of N_2_O emissions. This finding is consistent with an earlier study on the same site demonstrating that HS had the highest system-wide carbon footprint, attributable to poorer animal-performance than PP and higher fertilisation rates than WC (McAuliffe et al., 2018a).

### 4.3 Soil effect on N_2_O emissions

The PP treatment showed the highest soil TN concentration before treatment application and, in general, the increase in soil N concentration after treatment application was also more pronounced under this system. Given that less N_2_O was emitted from PP than HS (Section 4.2), the current findings suggest that the historic avoidance of ploughing on the PP farmlet may have caused a positive effect on soil N retention and resulting N_2_O emissions (Krol et al., 2016). This can be due to stability of the organic N that had not been disturbed, a hypothesis that is consistent with the higher levels of TC observed on the PP farmlet (Table 1). In addition, a forage C content under HS is thought to have promoted denitrification, resulting in larger emissions from multiple pathways. The increase in soil TN after treatment application was largely proportional to the amount of N applied, although the change was less pronounced under WC where no inorganic N was applied. At the same time, the rapid increase in herbage N concentration on WC after treatment application suggests a greater N uptake by the plants.

### 4.4 Microbial effect on N2O emissions

Significant pre-treatment differences in gene abundance between farmlets suggest that previous management (i.e. ploughing and reseeding of WC and HS) of these fields had conditioned the microbial populations. In this regard, the lowest total abundance of the *amoA* AOB gene detected on PP is in line with findings reported by Yao et al. (2013) who identified a lower *amoA* AOB abundance in semi-natural grassland compared to improved grassland. Across all farmlets and treatments, bacteria outnumbered archaea and fungi by three and one orders of magnitude, respectively. These results align with previous reports in grassland soils (Hayden et al., 2012; Mamet et al., 2017). Furthermore, *nosZ* genes were significantly more abundant on PP than WC and HS, suggesting a greater potential for N_2_O reduction, possibly driven by higher TC content from undisturbed soil. The average ratio across all treatments between genes involved in N_2_O production (*amoA* AOB+*amoA* AOA+*nirK*+*nirS*) and reduction (*nosZ*I+*nosZ*II) was the highest on HS (12.8), followed by WC (10.2) and PP (7.94) (Supplementary Table S2). These findings are consistent with the observed differences in cumulative N_2_O emissions between farmlets (Table 4).

The current results also show that the *amoA* AOB gene was more abundant than *amoA* AOA in the urine and dung treatments, which agrees with previous results from animal manure composts (Yamamoto et al. 2012) and soils treated with bovine urine and urea (Di et al., 2009, 2010). Increases in the abundance of nitrification genes may explain the observed N_2_O peaks during the dry period from SU and D treatments on all farmlets. This theory agrees with the low soil WFPS and the decrease in abundance of the *nirK* gene found after treatment application, collectively suggesting that nitrification was the main driving process of N_2_O production at this stage. The decrease in *nirK* abundance on the CON + N treatment after treatment application coincides with a smaller peak of N_2_O compared to SU and D treatments, as CON + N plots had less N to nitrify because only half of the fertiliser was in the form of NH_4_^+^. Overall, however, the general dominance of the *nirK* gene indicates that the follow-on peaks of N_2_O were due to denitrification, particularly as there were several rainfall events that increased WFPS up to 80 %.

### 4.5 Implications for grazing management

Collectively, the above results showcase strong interactions that connect soil, pasture, animal excreta and nitrifying/denitrifying microbial communities within grazing-based livestock production systems. Disregarding these interactions could result in overestimation or underestimation of the effect of adopting alternative farming strategies on N_2_O emissions; this can be seen, for example, from the discrepancy in EFs, both with respect to the absolute value for each system and the relative ratios amongst different systems (i.e. between the standardised treatment (SU) and system-specific (CU) natural urine) (Table 5). These observations, in turn, provide a strong case for system-based approaches to N_2_O measurements, which have seldom been employed by the literature to date. It is noteworthy, however, that experimentally-obtained EFs are necessarily aspatial, in the sense that they are measured in areas that are entirely covered by a urine patch or dung deposit. Naturally, this environment is not wholly representative of commercial grazing farms, as a large proportion of the area within any given field is left “untouched” for a long period of time (Dennis et al., 2011; Turner et al., 2016). While techniques exist to directly measure total N_2_O emissions at a field scale, e.g. the eddy covariance method, data acquired in this manner can neither be used to quantify source-specific EFs nor identify mechanistic pathways linking grazing management strategies and resultant emissions (Voglmeier et al., 2019).

To this end, Luo et al. (2010) carried out a review of grazing management strategies to reduce N_2_O emissions and provided an illuminating discussion. The authors considered numerous management options including soil management, fertilisation scheme, use of N-process inhibitors and adaptive animal rotation. Following an extensive search and reanalysis of quantitative data, a combination of multiple mitigation strategies, rather than sole reliance on the most promising one, was more likely to be effective: for example when adopting grazing (to avoid wet periods) was employed concurrently with soil amendments with slurry. This position is strongly in agreement with a recent life cycle assessment (LCA) study at the study site (the NWFP), which found that amongst all sources of uncertainty surrounding carbon footprints of grazing enterprises, uncertainty associated with N_2_O EFs is the largest, and therefore hedging the risk through a mixed strategy would be a sensible approach (Takahashi et al., 2019). In this context, other on-farm options that could contribute to the solution package include: light interception control through manipulation of sward structure (Congio et al., 2019); fertiliser selection to enhance pasture growth and thus the stocking rate (Louro et al., 2016); diet formulation to control N load and urination volume (Marsden et al., 2016); and direct control of grazing intensity (Tang et al., 2019).

As highlighted by Turner et al. (2016), subterranean processes to produce N_2_O are highly sensitive to spatial variability of environmental factors such as topography and soil conditions. While analysing impacts of these variables on overall emissions is beyond the scope of chamber-based trials including the present study, it is acknowledged that findings and EFs derived from these trials should not only facilitate policy debates (Section 1) but, ideally, also inform future farm management strategies that can adequately balance provision of nutritious food with environmental burdens (McAuliffe et al., 2018b; McAuliffe et al., 2020). For this purpose, the aforementioned insight into the merits and demerits of reseeding high sugar grasses (Section 4.2) are immediately communicable to the commercial farming community. Additionally, high-resolution information on animal movement and behaviour (de Weerd et al., 2015; Liu et al., 2015) and matched soil samples (Granger et al., 2017) would enable dynamically adaptive field management practices, e.g. mobile fencing (Freeman et al., 2019), to avoid creation of “N_2_O hotspots” (Turner et al., 2016). Both sets of data are currently being collected at the study site, which is uniquely designed to allow spatially detailed investigations (Orr et al., 2016; Takahashi et al., 2018).

## 5 Conclusions

N_2_O emissions were measured on three treatments (CU, SU and D) and two controls (CON + N and CON-N) under three pasture systems (PP, WC and HS) commonly observed in temperate regions. The results suggest that sward composition had a significant impact on animal excreta, most notably demonstrated by the lower urinary N excretion on HS compared to PP. However, the lower N found in HS cattle’s urine did not result in a net reduction in cumulative N_2_O emissions, with HS plots generally recording higher emissions than PP and WC. Differences in N_2_O emissions among pasture systems were successfully explained by the abundance ratios between genes known to be involved in N_2_O production and reduction. N_2_O emissions were mainly driven by nitrification in the first weeks after treatment application when the weather was relatively dry, whereas denitrification was dominant from this point onward when rainfall, and thus soil moisture, increased. Although the current findings cannot be immediately extrapolated to different climate and soil types, treatments were found to generate significantly higher emissions than controls, and EFs derived were largely within the IPCC 95 % range. Finally, the present results demonstrate the importance of evaluating EFs at a system scale, so that the feedback mechanisms linking soil, pasture, animals and microbiomes are appropriately considered.

## Declaration of Competing Interest

The authors declare that they have no known competing financial interests or personal relationships that could have appeared to influence the work reported in this paper.
