# Supplementary material for: Elucidating three-way interactions between soil, pasture and animals that regulate nitrous oxide emissions from temperate grazing systems
Source: Agric Ecosyst Environ. 2020 Sep 15;300:106978. doi: 10.1016/j.agee.2020.106978 (PMC7307388; doi:10.1016/j.agee.2020.106978)
Supplement: Supplementary file 1 [file mmc1.docx]

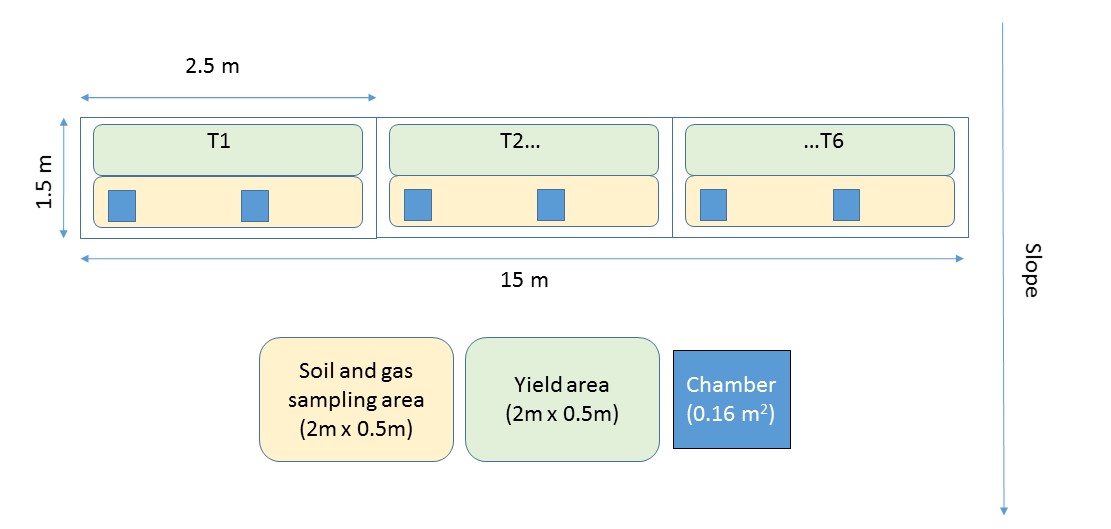


Supplementary figure 1. Plot and block design of the field experiment. For ease of display, treatments T3, T4 and T5 are omitted from the schematic but follow the same design as T1, T2 and T6.

Supplementary table 1. PCR primers and thermal cycling conditions used for quantification of different genes.

| **Primers** | **Sequence (5’ – 3’)** | **Thermal conditions** |
| --- | --- | --- |
| 16S rRNA^1^: 341F  534R | CCT ACG GGA GGC AGC AG  ATT ACC GCG GCT GCT GGC A | 95°C, 15 min, 1 cycle  95°C for 15 s, 60°C for 30 s, 72°C for 30 s, 80°C for 15 s, 40 cycles  95°C for 15 s, 60 to 95°C, 1 cycle |
| ***narG***^1^:  narG1960m2F  narG2050m2R | TAY GTS GGG CAG GAR AAA CTG  CGT AGA AGA AGC TGG TGC TGT T | 95°C, 15 min, 1 cycle  95°C for 15 s, 65 to 60°C for 30 s (-1°C by cycle), 72°C for 30 s, 80°C for 15 s, 6 cycles  95°C for 15 s, 60°C for 30 s, 72°C for 30 s, 80°C for 15 s, 40 cycles  95°C for 15 s, 60 to 95°C, 1 cycle |
| ***nirK***^2^:  nirK876  nirK1040 | ATY GGC GGV CAY GGC GA  GCC TCG ATC AGR TTR TGG TT | 95°C, 15 min, 1 cycle  95°C for 15 s, 63 to 58°C for 30 s (-1°C by cycle), 72°C for 30 s, 80°C for 15 s, 6 cycles  95°C for 15 s, 60°C for 30 s, 72°C for 30 s, 80°C for 15 s, 40 cycles  95°C for 15 s, 60 to 95°C, 1 cycle |
| ***nirS***^3^**:**  nirSCd3aFm  nirSR3cdm | AAC GYS AAG GAR ACS GG  GAS TTC GGR TGS GTC TTS AYG AA | 95°C, 15 min, 1 cycle  95°C for 15 s, 65 to 60°C for 30 s (-1°C by cycle), 72°C for 30 s, 80°C for 15 s, 6 cycles  95°C for 15 s, 60°C for 30 s, 72°C for 30 s, 80°C for 15 s, 40 cycles  95°C for 15 s, 60 to 95°C, 1 cycle |
| ***nosZ***^4^:  nosZ2F  nosZ2R´ | CGC RAC GGC AAS AAG GTS MSS GT  CAK RTG CAK SGC RTG GCA GAA | 95°C, 15 min, 1 cycle  95°C for 15 s, 65 to 60°C for 30 s (-1°C by cycle), 72°C for 30 s, 80°C for 15 s, 6 cycles  95°C for 15 s, 60°C for 30 s, 72°C for 30 s, 80°C for 15 s, 40 cycles  95°C for 15 s, 60 to 95°C, 1 cycle |
| ***amoA*(AOB)**^5^***:***  amoA-1F  amoA-2R | GGG GTT TCT ACT GGT GGT  CCC CTC KGS AAA GCC TTC TTC | 95°C, 10 min, 1 cycle  94°C for 45 s, 58°C for 45 s, 72°C for 45 s, 39 cycles  95°C for 15 s, 60°C for 30 s, to 95°C for 15 s, 1 cycle |
| ***amoA*(AOA)**  19F^6^  CrenamoA616r48x^7^ | ATG GTC TGG CTW AGA CG  GCC ATC CAB CKR TAN GTC CA | 95°C, 10 min, 1 cycle  94°C for 45 s, 55°C for 45 s, 72°C for 45 s, 39 cycles  95°C for 15 s, 60°C for 30 s, to 95°C for 15 s, 1 cycle |

^1^ Lopez-Gutierrez J.C., Henry S., Hallet S., Martin-Laurent F., Catrou, G., Philippot L. (2004) Quantification of a novel group of nitrate-reducing bacteria in the environment by real-time PCR. *J Microbiol Methods* **57**: 399-407.

^2^ Henry S., Baudouin, E., López-Gutiérrez, J.C., Martin-Laurent, F., Brauman, A., Philippot, L. (2004) Quantification of denitrifying bacteria in soils by *nirK* gene targeted real-time PCR. *J Microbiol Methods* **59**: 327-335. Corrigendum in *J Microbiol Methods*  **61** (2): 289-290

^3^ Throbäck N., Enwall K., Jarvis A., Hallin, S. (2004) Reassessing PCR primers targeting *nirS*, *nirK* and *nosZ* genes for community surveys of denitrifying bacteria with DGGE. *FEMS Microbiol Ecol* **49**: 401-417.

^4^ Henry S., Bru D., Stres B., Hallet S., Philippot, L. (2006) Quantitative detection of the *nosZ* gene, encoding nitrous oxide reductase, and comparison of the abundances of 16S rRNA, *narG, nirK*, and *nosZ* genes in soils. *Appl Environ Microbiol* **72**: 5181-5189.

^5^ Rotthauwe J.-H., Witzel K.-P., Liesack W. (1997) The ammonia monooxygenase structural gene *amoA* as a functional marker: Molecular fine-scale analysis of natural ammonia-oxidizing populations. *Appl Environ Microbiol* **63**: 4704-4712.

^6^ Leininger S, Urich T, Schloter M, Schwark L, Qi J, Nicol GW *et al.* (2006) Archaea predominate among ammonia-oxidizing prokaryotes in soils. *Nature* **442**: 806-809.

^7^ Leininger S., personal communication

Supplementary table 2. Ratio between genes involved in N_2_O production (amoA AOA + amoA AOB + nirK + nirS) and reduction (nosZI + nosZII genes) in a permanent pasture (PP), white clover/high sugar grass mix (WC) and high sugar grass monoculture (HS) farmlets. Time point 1= 4 days before treatment application; time point 2= 24 days after treatment application; time point 3= 126 days after treatment application.

| Treatment | Timepoint | Ratio N_2_O producers / N_2_O reducers | | |
| --- | --- | --- | --- | --- |
|  |  | Farmlet | | |
|  |  | PP | WC | HS |
| Inorganic N + dung (D) | 1 | 7.12 | 8.35 | 20.01 |
|  | 2 | 7.97 | 10.67 | 11.07 |
|  | 3 | 11.10 | 13.57 | 14.69 |
| Inorganic N + synthetic urine (SU) | 1 | 7.16 | 9.92 | 14.24 |
|  | 2 | 10.06 | 7.61 | 8.62 |
|  | 3 | 7.60 | 9.40 | 9.85 |
| Inorganic N+ urine (spring) (CU) | 1 | 7.37 | 9.26 | 18.26 |
|  | 2 | 10.59 | 6.42 | 7.87 |
|  | 3 | 6.52 | 14.64 | 15.55 |
| Inorganic N only (Control 2) (CON+N) | 1 | 10.08 | NA | 16.13 |
|  | 2 | 1.05 | NA | 0.86 |
|  | 3 | 7.97 | NA | 9.92 |
| No N (Control 1) (CON-N) | 1 | 9.13 | 8.61 | 13.51 |
|  | 2 | 7.47 | 9.25 | 14.09 |
|  | 3 | 7.94 | 14.53 | 17.90 |
