# Supplementary figures and images for: Elucidating three-way interactions between soil, pasture and animals that regulate nitrous oxide emissions from temperate grazing systems
Source: Agric Ecosyst Environ. 2020 Sep 15;300:106978. doi: 10.1016/j.agee.2020.106978 (PMC7307388; doi:10.1016/j.agee.2020.106978)

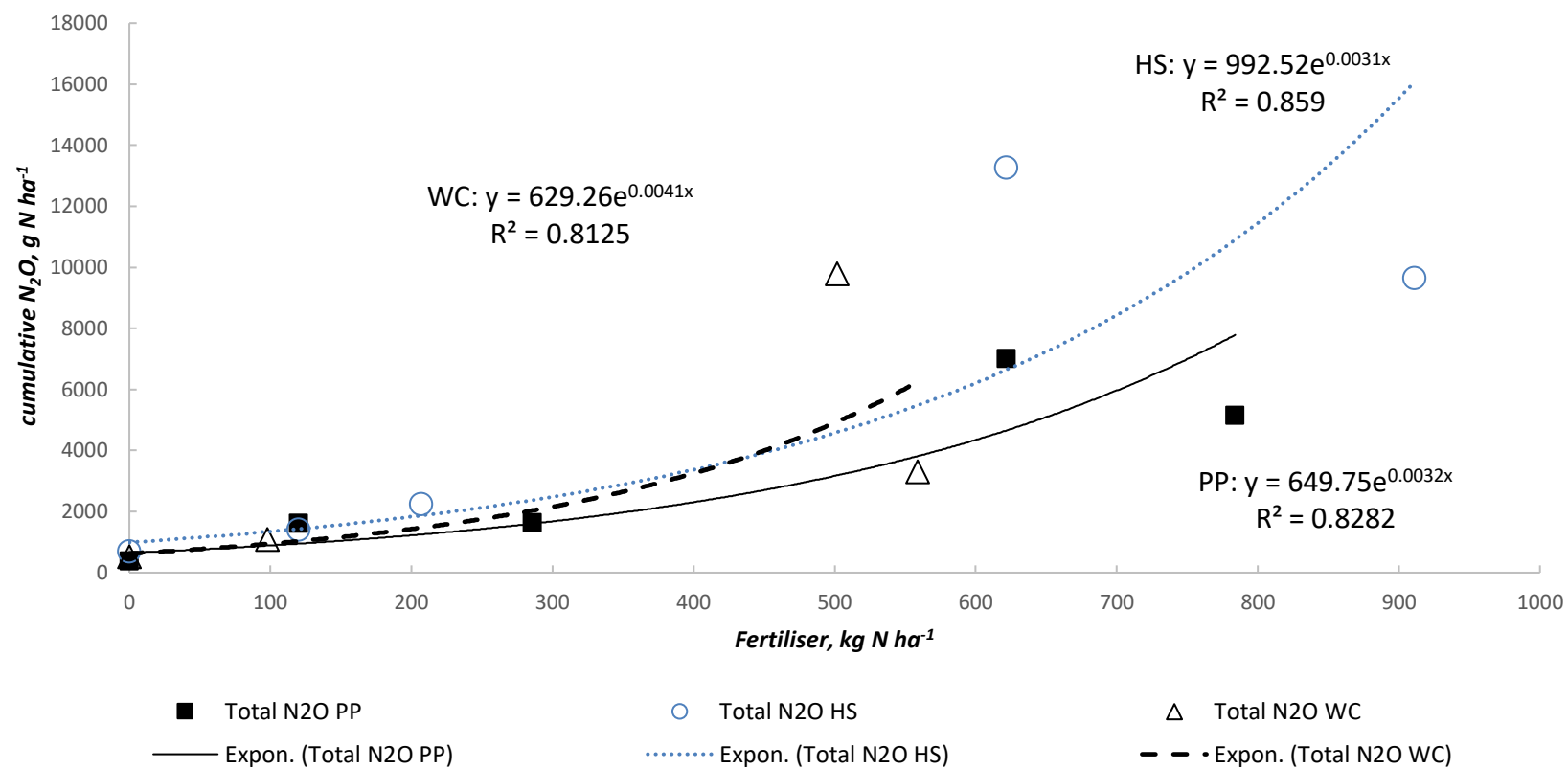

Supplement: Supplementary file 2 [file mmc2.pdf]
